# Tris(2,2′-bi-1*H*-imidazole-κ^2^ *N* ^3^,*N* ^3′^)­nickel(II) dinitrate *N*,*N*-dimethyl­formamide monosolvate

**DOI:** 10.1107/S1600536811043030

**Published:** 2011-10-22

**Authors:** Qi-Ming Qiu, Wei Yang, Zhong-Feng Li, Qiong-Hua Jin, Cun-Lin Zhang

**Affiliations:** a Department of Chemistry, Capital Normal University, Beijing 100048, People’s Republic of China; b Key Laboratory of Terahertz Optoelectronics, Ministry of Education, Department of Physics, Capital Normal University, Beijing 100048, People’s Republic of China

## Abstract

The reaction of nickel salts and 4,4′-bipyridine with 2,2′-bi­imidazole (H_2_biim) yielded the title complex, [Ni(C_6_H_6_N_4_)_3_](NO_3_)_2_·C_3_H_7_NO. The Ni^II^ atom is chelated by three H_2_biim ligands in a distorted octa­hedral coordination geometry. The two nitrate anions and one dimethyl­formamide (DMF) mol­ecule are not coordinated. The compound has a three-dimensional structure, formed by extensive hydrogen bonding between [Ni(H_2_biim)_3_]^2+^ cations and nitrate anions, each nitrate anion forming hydrogen bonds with an *R*_1_^2^(4) motif. The DMF molecule is disordered over three sets of sites, with occupancy ratios of 0.341 (16):0.350 (17):0.309 (19).

## Related literature

For related literature on the 2,2′-biimidazole ligand, see: Ding *et al.* (2005 ▶); Gruia *et al.* (2007 ▶); Martinez Lorente *et al.* (1995 ▶). For related structures, see: Dai *et al.* (2010 ▶); Jin *et al.* (2010 ▶); Yang *et al.* (2005 ▶).
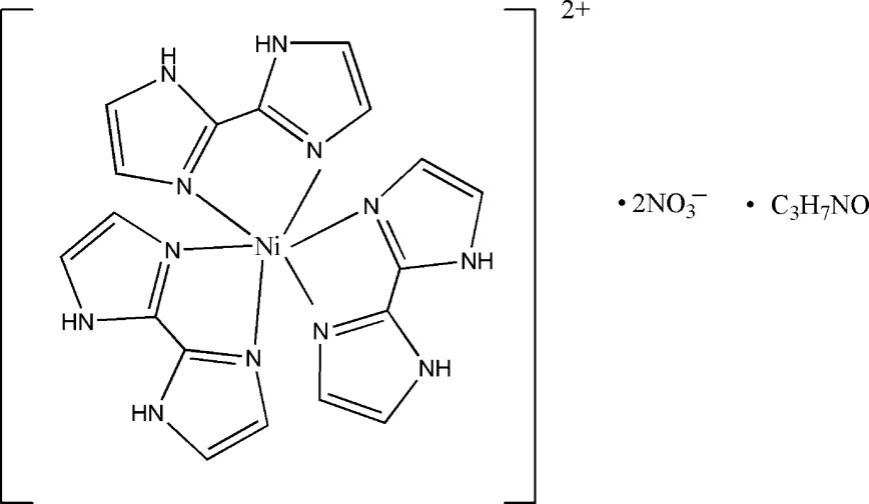


## Experimental

### 

#### Crystal data

[Ni(C_6_H_6_N_4_)_3_](NO_3_)_2_·C_3_H_7_NO
*M* *_r_* = 658.27
Monoclinic, 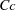

*a* = 12.2150 (11) Å
*b* = 20.864 (2) Å
*c* = 12.1080 (12) Å
β = 90.528 (1)°
*V* = 3085.6 (5) Å^3^
*Z* = 4
Mo *K*α radiation
μ = 0.69 mm^−1^
*T* = 298 K
0.36 × 0.19 × 0.12 mm

#### Data collection

Bruker SMART CCD area-detector diffractometer
Absorption correction: multi-scan (*SADABS*; Bruker, 2007 ▶) *T*_min_ = 0.788, *T*_max_ = 0.921
7496 measured reflections
4289 independent reflections
1969 reflections with *I* > 2σ(*I*)
*R*_int_ = 0.131

#### Refinement

*R*[*F* ^2^ > 2σ(*F* ^2^)] = 0.109
*wR*(*F* ^2^) = 0.276
*S* = 1.06
4289 reflections
437 parameters
3 restraints
H-atom parameters constrained
Δρ_max_ = 0.54 e Å^−3^
Δρ_min_ = −0.61 e Å^−3^
Absolute structure: Flack (1983 ▶), 2702 Friedel pairs
Flack parameter: 0.00 (6)

### 

Data collection: *SMART* (Bruker, 2007 ▶); cell refinement: *SAINT-Plus* (Bruker, 2007 ▶); data reduction: *SAINT-Plus*; program(s) used to solve structure: *SHELXS97* (Sheldrick, 2008 ▶); program(s) used to refine structure: *SHELXL97* (Sheldrick, 2008 ▶); molecular graphics: *SHELXTL* (Sheldrick, 2008 ▶); software used to prepare material for publication: *SHELXTL*.

## Supplementary Material

Crystal structure: contains datablock(s) global, I. DOI: 10.1107/S1600536811043030/zk2032sup1.cif

Structure factors: contains datablock(s) I. DOI: 10.1107/S1600536811043030/zk2032Isup2.hkl

Additional supplementary materials:  crystallographic information; 3D view; checkCIF report

## Figures and Tables

**Table 1:** Hydrogen-bond geometry (Å, °)

*D*—H⋯*A*	*D*—H	H⋯*A*	*D*⋯*A*	*D*—H⋯*A*
N2—H2⋯O5^i^	0.86	2.19	2.93 (2)	144
N2—H2⋯O4^i^	0.86	2.30	2.98 (2)	136
N4—H4⋯O1^ii^	0.86	1.91	2.77 (2)	176
N4—H4⋯O3^ii^	0.86	2.45	3.04 (3)	127
N6—H6⋯O6	0.86	2.05	2.90 (3)	171
N6—H6⋯O4	0.86	2.43	3.09 (2)	133
N8—H8⋯O2^iii^	0.86	2.09	2.82 (3)	141
N8—H8⋯O3^iii^	0.86	2.36	2.98 (2)	130
N10—H10⋯O5^iv^	0.86	2.17	2.98 (3)	156
N10—H10⋯O6^iv^	0.86	2.45	3.16 (2)	140
N12—H12⋯O2^v^	0.86	2.29	2.97 (2)	136

Symmetry codes: (i) 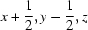; (ii) 
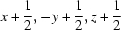
; (iii) 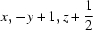; (iv) 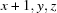; (v) 
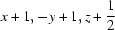
.

**Fig. 1.**
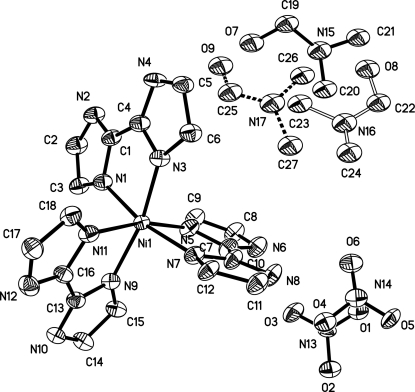
.1. The molecular entities of the title compound, showing the atom-numbering scheme and with displacement ellipsoids drawn at the 50% probability level.

**Fig. 2.**
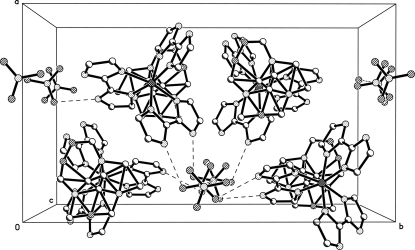
The three-dimensional structure of the title complex formed through intermolecular N—H···O hydrogen bonds.

## supplementary crystallographic information

### Comment

2,2'-Biimidazole is an excellent candidate for building a supramolecular structure involving directed hydrogen bonding interactions (Dai *et al.*, 2010; Ding *et al.*, 2005; Gruia *et al.*, 2007; Jin *et al.*, 2010; Yang *et al.*, 2005). This versatile molecule can act as a non-deprotonated, mono-deprotonated or bis-deprotonated ligand (Martinez Lorente *et al.*, 1995). Furthermore, the uncoordinated N—H groups in H_2_biim can participate in various patterns of hydrogen bonds with other acceptors. This may provides useful information to understand the complicated process in biological systems (Gruia *et al.*, 2007; Ding *et al.*, 2005). Herein we report the compound [Ni(H_2_biim)_3_(NO_3_)_2_].C_3_H_7_NO.

The asymmetry unit of the title compound consists of one [Ni(H_2_biim)_3_]^2+^ cation,two nitrate anions and one free *N*,*N*-dimethyl formamide molecule(Fig.1). The complex is monoclinic in *Cc* space group. In the title complex, the metal center allows the formation of a distorted octahedral geometry with six nitrogen atoms of three chelating H_2_biim ligands. The compound contains three bidentate H_2_biim ligands which provide six external N—H groups, which form hyfrogen bonds with the O atoms of NO_3_^-^ anions to generate an extended hydrogen-bonded three-dimensional structure (Fig.2).

In [Ni(H_2_biim)_3_(NO_3_)_2_].C_3_H_7_NO, the Ni—N bond lengths are in the range 2.005 (2)–2.106 (2) Å, which agree with those in the compound [Ni(C_6_H_6_N_4_)_3_](C_8_H_4_O_4_) (C_8_H_4_O_4_ = phthalate dianions)(Yang *et al.*, 2005). The distorted N—Ni—N bite angles of H_2_biim ligands [N3—Ni1—N1 = 81.1 (7)°, N5—Ni1—N7 = 81.6 (8)°, N9—Ni1—N11 = 80.2 (8)°] agree with the corresponding angles for [Ni(H_2_biim)_3_]^2+^ complexes (Yang *et al.*, 2005). The hydrogen bonds are formed between the N—H donors of H_2_biim and the oxygen atoms from NO_3_^-^ (O4 and O5), with d(N2···O4) = 2.979 Å, d(N2···O5) = 2.930 Å, N2—H2···O4 =136.28° and N2—H2···O5 = 144.29°.

It is interesting that the solvated DMF did not come from the starting materials. This phenomena also appeared in other similar reactions of H_2_biim with metal salts in the solvent CH_3_OH and H_2_O.

### Experimental

The title complex has been prepared by adding 4,4'-bipyridine (0.2 mmol) and H_2_biim (0.4 mmol) into a stirred CH_3_OH (5 mL) and H_2_O (5 mL) containing Ni(NO_3_)_2_.6H_2_O (0.2 mmol). The mixture was refluxed for 30 min and then allowed to cool to ambient temperature. The filtrate was evaporated slowly at room temperature for several weeks to yield purple crystalline products.

### Refinement

Metal atom centers were located from the E-maps and other non-hydrogen atoms were located in successive difference Fourier syntheses. The final refinements were performed by full matrix least-squares methods with anisotropic thermal parameters for non-hydrogen atoms on F^2^.

The final refinements were performed with isotropic thermal parameters. All hydrogen atoms were located in the calculated sites and included in the final refinement in the riding model approximation with displacement parameters derived from the parent atoms to which they were bonded.

Data collection: *SMART* (Bruker, 2007); cell refinement: SAINTPlus (Bruker, 2007); data reduction: *SAINT-Plus*; program(*s*) used to solve structure: *SHELXS97* (Sheldrick, 2008); program(*s*) used to refine structure: *SHELXL97* (Sheldrick, 2008); molecular graphics: *SHELXTL* (Sheldrick, 2008); software used to prepare material for publication: *SHELXTL*.

### Crystal data

**Table:**

[Ni(C_6_H_6_N_4_)_3_](NO_3_)_2_·C_3_H_7_NO	*F*(000) = 1360
*M**_r_* = 658.27	*D*_x_ = 1.417 Mg m^−^^3^
Monoclinic, *C**c*	Mo *K*α radiation, λ = 0.71073 Å
*a* = 12.2150 (11) Å	Cell parameters from 1160 reflections
*b* = 20.864 (2) Å	θ = 2.5–17.3°
*c* = 12.1080 (12) Å	µ = 0.69 mm^−^^1^
β = 90.528 (1)°	*T* = 298 K
*V* = 3085.6 (5) Å^3^	Block, purple
*Z* = 4	0.36 × 0.19 × 0.12 mm

### Data collection

**Table:**

Bruker SMART CCD area-detector diffractometer	4289 independent reflections
Radiation source: fine-focus sealed tube	1969 reflections with *I* > 2σ(*I*)
graphite	*R*_int_ = 0.131
phi and ω scans	θ_max_ = 25.0°, θ_min_ = 2.6°
Absorption correction: multi-scan (*SADABS*; Bruker, 2007)	*h* = −14→14
*T*_min_ = 0.788, *T*_max_ = 0.921	*k* = −23→24
7496 measured reflections	*l* = −14→13

### Refinement

**Table:**

Refinement on *F*^2^	Secondary atom site location: difference Fourier map
Least-squares matrix: full	Hydrogen site location: inferred from neighbouring sites
*R*[*F*^2^ > 2σ(*F*^2^)] = 0.109	H-atom parameters constrained
*wR*(*F*^2^) = 0.276	*w* = 1/[σ^2^(*F*_o_^2^) + (0.117*P*)^2^] where *P* = (*F*_o_^2^ + 2*F*_c_^2^)/3
*S* = 1.06	(Δ/σ)_max_ = 0.001
4289 reflections	Δρ_max_ = 0.54 e Å^−^^3^
437 parameters	Δρ_min_ = −0.61 e Å^−^^3^
3 restraints	Absolute structure: Flack (1983), 1549 Friedel pairs
Primary atom site location: structure-invariant direct methods	Flack parameter: 0.00 (6)

### Special details

**Table:**

Geometry. All e.s.d.'s (except the e.s.d. in the dihedral angle between two l.s. planes) are estimated using the full covariance matrix. The cell e.s.d.'s are taken into account individually in the estimation of e.s.d.'s in distances, angles and torsion angles; correlations between e.s.d.'s in cell parameters are only used when they are defined by crystal symmetry. An approximate (isotropic) treatment of cell e.s.d.'s is used for estimating e.s.d.'s involving l.s. planes.
Refinement. Refinement of *F*^2^ against ALL reflections. The weighted *R*-factor *wR* and goodness of fit *S* are based on *F*^2^, conventional *R*-factors *R* are based on *F*, with *F* set to zero for negative *F*^2^. The threshold expression of *F*^2^ > σ(*F*^2^) is used only for calculating *R*-factors(gt) *etc*. and is not relevant to the choice of reflections for refinement. *R*-factors based on *F*^2^ are statistically about twice as large as those based on *F*, and *R*- factors based on ALL data will be even larger.

### Fractional atomic coordinates and isotropic or equivalent isotropic displacement parameters (Å^2^)

**Table:**

	*x*	*y*	*z*	*U*_iso_*/*U*_eq_	Occ. (<1)
Ni1	0.6556 (2)	0.33319 (8)	0.3945 (2)	0.0423 (5)	
N1	0.7111 (15)	0.2490 (8)	0.3170 (16)	0.065 (5)	
N2	0.6946 (14)	0.1433 (8)	0.3119 (16)	0.075 (5)	
H2	0.6816	0.1045	0.3317	0.090*	
N3	0.6035 (15)	0.2688 (8)	0.5051 (14)	0.062 (5)	
N4	0.5823 (19)	0.1662 (7)	0.5541 (19)	0.072 (6)	
H4	0.5800	0.1251	0.5481	0.086*	
N5	0.5220 (19)	0.3337 (7)	0.2926 (18)	0.068 (6)	
N6	0.3559 (17)	0.3726 (9)	0.2553 (18)	0.085 (6)	
H6	0.2946	0.3922	0.2640	0.102*	
N7	0.5692 (17)	0.4055 (8)	0.4700 (15)	0.067 (5)	
N8	0.4052 (15)	0.4534 (8)	0.4806 (16)	0.079 (6)	
H8	0.3384	0.4638	0.4666	0.095*	
N9	0.7373 (16)	0.3892 (8)	0.2801 (16)	0.065 (5)	
N10	0.8848 (15)	0.4446 (7)	0.2328 (16)	0.067 (5)	
H10	0.9503	0.4596	0.2342	0.081*	
N11	0.7983 (16)	0.3544 (8)	0.4856 (16)	0.064 (5)	
N12	0.9522 (14)	0.4110 (8)	0.4904 (17)	0.076 (5)	
H12	1.0047	0.4360	0.4712	0.091*	
N13	0.161 (2)	0.4854 (9)	0.0305 (15)	0.072 (5)	
N14	0.154 (2)	0.4795 (11)	0.2929 (17)	0.081 (6)	
N15	0.102 (8)	0.110 (4)	0.383 (5)	0.103 (14)	0.341 (16)
N16	0.103 (6)	0.237 (4)	0.409 (5)	0.103 (14)	0.350 (17)
N17	0.266 (9)	0.186 (4)	0.395 (7)	0.103 (14)	0.309 (19)
O1	0.0657 (16)	0.4662 (7)	0.0412 (16)	0.102 (5)	
O2	0.1804 (15)	0.5453 (8)	0.0304 (14)	0.093 (5)	
O3	0.2365 (15)	0.4472 (8)	0.0272 (12)	0.098 (5)	
O4	0.2461 (15)	0.5046 (7)	0.2861 (11)	0.081 (4)	
O5	0.0835 (15)	0.5192 (7)	0.2951 (14)	0.091 (5)	
O6	0.1376 (15)	0.4256 (8)	0.2796 (14)	0.097 (5)	
O7	0.266 (6)	0.077 (3)	0.364 (4)	0.104 (11)	0.341 (16)
O8	−0.002 (5)	0.155 (3)	0.400 (4)	0.104 (11)	0.350 (17)
O9	0.358 (5)	0.104 (3)	0.352 (5)	0.104 (11)	0.309 (19)
C1	0.6771 (18)	0.1972 (8)	0.3734 (19)	0.066 (6)	
C2	0.737 (3)	0.1628 (11)	0.212 (3)	0.089 (9)	
H2B	0.7517	0.1368	0.1521	0.107*	
C3	0.7530 (18)	0.2277 (10)	0.219 (2)	0.071 (6)	
H3	0.7867	0.2530	0.1663	0.086*	
C4	0.6245 (18)	0.2077 (10)	0.4790 (18)	0.068 (6)	
C5	0.5439 (16)	0.2016 (10)	0.6411 (19)	0.074 (6)	
H5	0.5196	0.1846	0.7075	0.089*	
C6	0.5468 (17)	0.2652 (10)	0.6160 (18)	0.074 (6)	
H6A	0.5199	0.2991	0.6577	0.089*	
C7	0.446 (2)	0.3781 (10)	0.321 (2)	0.075 (7)	
C8	0.380 (2)	0.3300 (10)	0.173 (2)	0.088 (7)	
H8A	0.3379	0.3212	0.1104	0.106*	
C9	0.479 (2)	0.3029 (11)	0.2000 (19)	0.083 (7)	
H9	0.5120	0.2692	0.1626	0.099*	
C10	0.469 (2)	0.4126 (10)	0.422 (2)	0.073 (7)	
C11	0.469 (2)	0.4745 (10)	0.567 (2)	0.080 (7)	
H11	0.4484	0.5041	0.6199	0.096*	
C12	0.5682 (19)	0.4447 (9)	0.5607 (19)	0.071 (6)	
H12A	0.6259	0.4501	0.6105	0.085*	
C13	0.833 (2)	0.4131 (9)	0.317 (2)	0.064 (6)	
C14	0.8125 (19)	0.4477 (10)	0.147 (2)	0.082 (6)	
H14	0.8234	0.4689	0.0801	0.099*	
C15	0.7209 (19)	0.4138 (9)	0.1757 (19)	0.073 (6)	
H15	0.6584	0.4085	0.1324	0.088*	
C16	0.8647 (18)	0.3931 (10)	0.427 (2)	0.062 (6)	
C17	0.9403 (19)	0.3817 (11)	0.591 (2)	0.077 (7)	
H17	0.9886	0.3849	0.6504	0.092*	
C18	0.8452 (18)	0.3468 (9)	0.5885 (19)	0.071 (6)	
H18	0.8174	0.3224	0.6461	0.086*	
C19	0.176 (8)	0.065 (3)	0.405 (7)	0.097 (15)	0.341 (16)
H19	0.1619	0.0286	0.4466	0.117*	0.341 (16)
C20	0.126 (11)	0.178 (6)	0.403 (10)	0.10 (2)	0.341 (16)
H20A	0.1347	0.1849	0.4811	0.143*	0.341 (16)
H20B	0.0657	0.2034	0.3760	0.143*	0.341 (16)
H20C	0.1914	0.1899	0.3657	0.143*	0.341 (16)
C21	−0.015 (7)	0.094 (4)	0.373 (6)	0.100 (14)	0.341 (16)
H21A	−0.0309	0.0804	0.2988	0.150*	0.341 (16)
H21B	−0.0573	0.1315	0.3897	0.150*	0.341 (16)
H21C	−0.0325	0.0604	0.4236	0.150*	0.341 (16)
C22	0.001 (7)	0.214 (4)	0.413 (6)	0.097 (15)	0.350 (17)
H22	−0.0604	0.2396	0.4246	0.117*	0.350 (17)
C23	0.195 (11)	0.192 (7)	0.390 (9)	0.10 (2)	0.350 (17)
H23A	0.1675	0.1534	0.3550	0.143*	0.350 (17)
H23B	0.2286	0.1809	0.4591	0.143*	0.350 (17)
H23C	0.2477	0.2116	0.3427	0.143*	0.350 (17)
C24	0.135 (6)	0.296 (3)	0.467 (6)	0.100 (14)	0.350 (17)
H24A	0.0830	0.3288	0.4518	0.150*	0.350 (17)
H24B	0.2063	0.3088	0.4425	0.150*	0.350 (17)
H24C	0.1380	0.2875	0.5452	0.150*	0.350 (17)
C25	0.364 (9)	0.159 (5)	0.392 (7)	0.097 (15)	0.309 (19)
H25	0.4286	0.1781	0.4163	0.117*	0.309 (19)
C26	0.166 (12)	0.147 (6)	0.386 (12)	0.10 (2)	0.309 (19)
H26A	0.1795	0.1111	0.3375	0.143*	0.309 (19)
H26B	0.1077	0.1725	0.3559	0.143*	0.309 (19)
H26C	0.1463	0.1313	0.4574	0.143*	0.309 (19)
C27	0.251 (8)	0.256 (4)	0.397 (6)	0.100 (14)	0.309 (19)
H27A	0.2538	0.2709	0.4717	0.150*	0.309 (19)
H27B	0.1813	0.2663	0.3645	0.150*	0.309 (19)
H27C	0.3081	0.2760	0.3552	0.150*	0.309 (19)

### Atomic displacement parameters (Å^2^)

**Table:**

	*U*^11^	*U*^22^	*U*^33^	*U*^12^	*U*^13^	*U*^23^
Ni1	0.0549 (11)	0.0431 (10)	0.0289 (10)	0.0010 (13)	−0.0019 (7)	−0.0009 (13)
N1	0.083 (13)	0.054 (10)	0.059 (13)	−0.001 (9)	0.002 (9)	−0.003 (10)
N2	0.113 (15)	0.051 (9)	0.061 (12)	0.011 (8)	0.017 (10)	−0.010 (9)
N3	0.084 (12)	0.052 (10)	0.051 (12)	−0.004 (8)	0.005 (9)	−0.002 (8)
N4	0.108 (18)	0.052 (11)	0.055 (14)	−0.008 (10)	0.012 (12)	0.015 (10)
N5	0.084 (13)	0.059 (10)	0.061 (13)	0.010 (10)	−0.014 (10)	−0.009 (9)
N6	0.096 (15)	0.088 (14)	0.072 (15)	0.023 (12)	−0.014 (13)	−0.004 (11)
N7	0.084 (15)	0.058 (10)	0.058 (13)	0.007 (9)	0.001 (10)	−0.002 (9)
N8	0.094 (14)	0.077 (11)	0.067 (14)	0.039 (10)	0.001 (11)	−0.012 (10)
N9	0.073 (13)	0.056 (10)	0.066 (14)	−0.003 (8)	−0.001 (10)	0.004 (9)
N10	0.069 (13)	0.062 (10)	0.071 (14)	−0.014 (9)	0.005 (10)	0.006 (10)
N11	0.076 (14)	0.053 (9)	0.061 (13)	−0.004 (9)	−0.014 (11)	0.000 (9)
N12	0.070 (12)	0.075 (12)	0.084 (15)	−0.018 (9)	−0.011 (10)	−0.008 (11)
N13	0.096 (17)	0.057 (12)	0.064 (13)	−0.004 (12)	0.018 (11)	0.023 (9)
N14	0.101 (18)	0.073 (15)	0.071 (15)	−0.011 (15)	−0.015 (12)	−0.006 (12)
N15	0.14 (4)	0.11 (3)	0.05 (2)	0.01 (3)	−0.01 (3)	0.03 (2)
N16	0.14 (4)	0.11 (3)	0.05 (2)	0.01 (3)	−0.01 (3)	0.03 (2)
N17	0.14 (4)	0.11 (3)	0.05 (2)	0.01 (3)	−0.01 (3)	0.03 (2)
O1	0.114 (15)	0.084 (11)	0.107 (15)	0.007 (10)	0.011 (11)	0.028 (10)
O2	0.121 (14)	0.074 (10)	0.085 (12)	−0.001 (9)	−0.003 (10)	0.013 (9)
O3	0.131 (14)	0.097 (11)	0.065 (11)	0.043 (11)	−0.005 (9)	0.027 (8)
O4	0.107 (13)	0.083 (10)	0.054 (10)	0.006 (10)	−0.009 (9)	0.011 (8)
O5	0.111 (13)	0.066 (9)	0.094 (14)	0.036 (9)	0.010 (10)	−0.012 (8)
O6	0.124 (14)	0.088 (11)	0.080 (13)	−0.002 (11)	−0.006 (10)	0.015 (10)
O7	0.14 (4)	0.11 (3)	0.056 (18)	0.00 (2)	−0.01 (2)	0.032 (18)
O8	0.14 (4)	0.11 (3)	0.056 (18)	0.00 (2)	−0.01 (2)	0.032 (18)
O9	0.14 (4)	0.11 (3)	0.056 (18)	0.00 (2)	−0.01 (2)	0.032 (18)
C1	0.092 (19)	0.050 (10)	0.057 (18)	0.008 (11)	0.004 (13)	−0.002 (11)
C2	0.12 (3)	0.072 (18)	0.07 (2)	0.011 (13)	0.013 (17)	−0.006 (14)
C3	0.100 (17)	0.058 (14)	0.056 (16)	0.010 (11)	0.008 (12)	−0.006 (11)
C4	0.089 (16)	0.061 (14)	0.054 (16)	−0.002 (11)	0.002 (12)	0.004 (12)
C5	0.101 (17)	0.061 (13)	0.060 (15)	0.004 (11)	0.030 (12)	0.008 (11)
C6	0.101 (17)	0.063 (13)	0.058 (15)	0.001 (11)	0.019 (12)	0.000 (10)
C7	0.084 (19)	0.069 (14)	0.071 (18)	0.011 (13)	−0.008 (14)	−0.014 (12)
C8	0.101 (19)	0.083 (15)	0.081 (17)	0.022 (15)	−0.014 (14)	−0.007 (15)
C9	0.096 (18)	0.078 (15)	0.074 (18)	0.017 (13)	−0.009 (14)	−0.005 (13)
C10	0.086 (18)	0.067 (13)	0.066 (17)	0.026 (12)	0.008 (13)	−0.006 (12)
C11	0.10 (2)	0.072 (14)	0.066 (17)	0.024 (13)	0.008 (14)	−0.010 (12)
C12	0.098 (17)	0.056 (12)	0.059 (15)	0.027 (12)	0.004 (12)	−0.010 (11)
C13	0.073 (16)	0.053 (11)	0.066 (17)	−0.004 (11)	0.004 (13)	−0.004 (11)
C14	0.088 (17)	0.082 (15)	0.077 (18)	−0.022 (13)	0.007 (14)	0.004 (13)
C15	0.090 (16)	0.064 (13)	0.066 (16)	−0.015 (11)	0.001 (12)	0.000 (11)
C16	0.067 (15)	0.056 (13)	0.064 (16)	−0.012 (10)	−0.002 (12)	−0.006 (11)
C17	0.073 (16)	0.075 (14)	0.083 (19)	−0.012 (12)	−0.018 (13)	−0.011 (14)
C18	0.078 (16)	0.063 (12)	0.073 (16)	0.001 (11)	−0.017 (12)	−0.002 (11)
C19	0.14 (5)	0.10 (3)	0.05 (3)	0.01 (3)	−0.01 (3)	0.03 (3)
C20	0.14 (7)	0.11 (6)	0.04 (3)	0.01 (5)	−0.02 (4)	0.04 (4)
C21	0.14 (4)	0.11 (3)	0.05 (3)	0.01 (3)	−0.01 (2)	0.03 (3)
C22	0.14 (5)	0.10 (3)	0.05 (3)	0.01 (3)	−0.01 (3)	0.03 (3)
C23	0.14 (7)	0.11 (6)	0.04 (3)	0.01 (5)	−0.02 (4)	0.04 (4)
C24	0.14 (4)	0.11 (3)	0.05 (3)	0.01 (3)	−0.01 (2)	0.03 (3)
C25	0.14 (5)	0.10 (3)	0.05 (3)	0.01 (3)	−0.01 (3)	0.03 (3)
C26	0.14 (7)	0.11 (6)	0.04 (3)	0.01 (5)	−0.02 (4)	0.04 (4)
C27	0.14 (4)	0.11 (3)	0.05 (3)	0.01 (3)	−0.01 (2)	0.03 (3)

### Geometric parameters (Å, °)

**Table:**

Ni1—N3	2.005 (18)	N17—C25	1.32 (11)
Ni1—N5	2.04 (2)	N17—C27	1.47 (11)
Ni1—N7	2.060 (19)	N17—C26	1.48 (16)
Ni1—N9	2.075 (19)	O7—C19	1.24 (10)
Ni1—N11	2.101 (18)	O8—C22	1.25 (8)
Ni1—N1	2.106 (18)	O9—C25	1.24 (9)
N1—C1	1.35 (2)	C1—C4	1.45 (3)
N1—C3	1.37 (3)	C2—C3	1.37 (3)
N2—C1	1.37 (2)	C2—H2B	0.9300
N2—C2	1.38 (4)	C3—H3	0.9300
N2—H2	0.8600	C5—C6	1.36 (3)
N3—C4	1.34 (2)	C5—H5	0.9300
N3—C6	1.52 (3)	C6—H6A	0.9300
N4—C4	1.36 (3)	C7—C10	1.44 (3)
N4—C5	1.37 (3)	C8—C9	1.37 (3)
N4—H4	0.8600	C8—H8A	0.9300
N5—C7	1.35 (3)	C9—H9	0.9300
N5—C9	1.39 (3)	C11—C12	1.36 (3)
N6—C7	1.36 (3)	C11—H11	0.9300
N6—C8	1.37 (3)	C12—H12A	0.9300
N6—H6	0.8600	C13—C16	1.45 (3)
N7—C10	1.36 (3)	C14—C15	1.37 (3)
N7—C12	1.37 (3)	C14—H14	0.9300
N8—C10	1.36 (3)	C15—H15	0.9300
N8—C11	1.37 (3)	C17—C18	1.37 (3)
N8—H8	0.8600	C17—H17	0.9300
N9—C13	1.35 (3)	C18—H18	0.9300
N9—C15	1.38 (3)	C19—H19	0.9300
N10—C14	1.36 (3)	C20—H20A	0.9600
N10—C13	1.37 (3)	C20—H20B	0.9600
N10—H10	0.8600	C20—H20C	0.9600
N11—C16	1.35 (3)	C21—H21A	0.9600
N11—C18	1.38 (3)	C21—H21B	0.9600
N12—C16	1.36 (2)	C21—H21C	0.9600
N12—C17	1.37 (3)	C22—H22	0.9300
N12—H12	0.8600	C23—H23A	0.9600
N13—O3	1.22 (2)	C23—H23B	0.9600
N13—O1	1.23 (2)	C23—H23C	0.9600
N13—O2	1.27 (2)	C24—H24A	0.9600
N14—O6	1.15 (2)	C24—H24B	0.9600
N14—O5	1.20 (2)	C24—H24C	0.9600
N14—O4	1.24 (3)	C25—H25	0.9300
N15—C19	1.32 (8)	C26—H26A	0.9600
N15—C21	1.47 (11)	C26—H26B	0.9600
N15—C20	1.48 (12)	C26—H26C	0.9600
N16—C22	1.33 (9)	C27—H27A	0.9600
N16—C24	1.47 (9)	C27—H27B	0.9600
N16—C23	1.48 (13)	C27—H27C	0.9600
			
N3—Ni1—N5	98.6 (8)	N2—C2—H2B	126.5
N3—Ni1—N7	91.5 (8)	N1—C3—C2	109 (3)
N5—Ni1—N7	81.6 (8)	N1—C3—H3	125.7
N3—Ni1—N9	168.8 (8)	C2—C3—H3	125.7
N5—Ni1—N9	88.9 (8)	N3—C4—N4	112 (2)
N7—Ni1—N9	97.8 (6)	N3—C4—C1	116.0 (19)
N3—Ni1—N11	93.3 (7)	N4—C4—C1	132 (2)
N5—Ni1—N11	166.6 (7)	C6—C5—N4	110 (2)
N7—Ni1—N11	92.2 (8)	C6—C5—H5	125.0
N9—Ni1—N11	80.2 (8)	N4—C5—H5	125.0
N3—Ni1—N1	81.1 (7)	C5—C6—N3	105.0 (18)
N5—Ni1—N1	89.7 (7)	C5—C6—H6A	127.5
N7—Ni1—N1	167.6 (8)	N3—C6—H6A	127.5
N9—Ni1—N1	90.8 (7)	N5—C7—N6	110 (2)
N11—Ni1—N1	98.1 (7)	N5—C7—C10	115 (2)
C1—N1—C3	107.2 (17)	N6—C7—C10	133 (3)
C1—N1—Ni1	110.0 (15)	C9—C8—N6	107 (2)
C3—N1—Ni1	141.4 (15)	C9—C8—H8A	126.6
C1—N2—C2	107.2 (17)	N6—C8—H8A	126.6
C1—N2—H2	126.4	C8—C9—N5	109 (2)
C2—N2—H2	126.4	C8—C9—H9	125.4
C4—N3—C6	104.5 (18)	N5—C9—H9	125.4
C4—N3—Ni1	114.7 (15)	N7—C10—N8	111 (2)
C6—N3—Ni1	140.7 (13)	N7—C10—C7	118 (2)
C4—N4—C5	107.7 (16)	N8—C10—C7	130 (2)
C4—N4—H4	126.1	C12—C11—N8	108 (2)
C5—N4—H4	126.1	C12—C11—H11	125.8
C7—N5—C9	105 (2)	N8—C11—H11	125.8
C7—N5—Ni1	113.4 (16)	C11—C12—N7	109 (2)
C9—N5—Ni1	141.2 (17)	C11—C12—H12A	125.4
C7—N6—C8	107 (2)	N7—C12—H12A	125.4
C7—N6—H6	126.3	N9—C13—N10	110 (2)
C8—N6—H6	126.3	N9—C13—C16	115 (2)
C10—N7—C12	105.4 (19)	N10—C13—C16	135 (2)
C10—N7—Ni1	110.6 (15)	N10—C14—C15	108 (2)
C12—N7—Ni1	143.3 (16)	N10—C14—H14	126.2
C10—N8—C11	106 (2)	C15—C14—H14	126.2
C10—N8—H8	127.1	C14—C15—N9	108 (2)
C11—N8—H8	127.1	C14—C15—H15	125.8
C13—N9—C15	106.4 (19)	N9—C15—H15	125.8
C13—N9—Ni1	114.3 (16)	N11—C16—N12	110 (2)
C15—N9—Ni1	139.1 (16)	N11—C16—C13	120 (2)
C14—N10—C13	107.1 (19)	N12—C16—C13	130 (2)
C14—N10—H10	126.5	N12—C17—C18	108 (2)
C13—N10—H10	126.5	N12—C17—H17	125.8
C16—N11—C18	107.3 (19)	C18—C17—H17	125.8
C16—N11—Ni1	110.4 (15)	C17—C18—N11	108 (2)
C18—N11—Ni1	142.0 (17)	C17—C18—H18	126.2
C16—N12—C17	107 (2)	N11—C18—H18	126.2
C16—N12—H12	126.6	O7—C19—N15	113 (8)
C17—N12—H12	126.6	O7—C19—H19	123.6
O3—N13—O1	120.2 (19)	N15—C19—H19	123.6
O3—N13—O2	120 (2)	O8—C22—N16	112 (8)
O1—N13—O2	120 (2)	O8—C22—H22	124.0
O6—N14—O5	124 (3)	N16—C22—H22	124.0
O6—N14—O4	124 (3)	N16—C23—H23A	109.5
O5—N14—O4	111 (2)	N16—C23—H23B	109.5
C19—N15—C21	122 (8)	H23A—C23—H23B	109.5
C19—N15—C20	121 (9)	N16—C23—H23C	109.5
C21—N15—C20	114 (8)	H23A—C23—H23C	109.5
C22—N16—C24	122 (8)	H23B—C23—H23C	109.5
C22—N16—C23	120 (9)	N16—C24—H24A	109.5
C24—N16—C23	114 (8)	N16—C24—H24B	109.5
C25—N17—C27	123 (10)	H24A—C24—H24B	109.5
C25—N17—C26	120 (10)	N16—C24—H24C	109.5
C27—N17—C26	116 (10)	H24A—C24—H24C	109.5
N1—C1—N2	109.6 (18)	H24B—C24—H24C	109.5
N1—C1—C4	117.7 (18)	O9—C25—N17	111 (10)
N2—C1—C4	132.5 (18)	O9—C25—H25	124.5
C3—C2—N2	107 (3)	N17—C25—H25	124.5
C3—C2—H2B	126.5		
			
N3—Ni1—N1—C1	4.9 (14)	N2—C2—C3—N1	−6(3)
N5—Ni1—N1—C1	−93.9 (15)	C6—N3—C4—N4	4(2)
N7—Ni1—N1—C1	−49 (4)	Ni1—N3—C4—N4	−176.8 (15)
N9—Ni1—N1—C1	177.2 (14)	C6—N3—C4—C1	178.6 (17)
N11—Ni1—N1—C1	97.0 (14)	Ni1—N3—C4—C1	−3(2)
N3—Ni1—N1—C3	169 (2)	C5—N4—C4—N3	−8(3)
N5—Ni1—N1—C3	70 (2)	C5—N4—C4—C1	179 (2)
N7—Ni1—N1—C3	115 (4)	N1—C1—C4—N3	7(3)
N9—Ni1—N1—C3	−19 (2)	N2—C1—C4—N3	−168 (2)
N11—Ni1—N1—C3	−99 (2)	N1—C1—C4—N4	−180 (2)
N5—Ni1—N3—C4	87.1 (16)	N2—C1—C4—N4	5(4)
N7—Ni1—N3—C4	168.8 (15)	C4—N4—C5—C6	9(3)
N9—Ni1—N3—C4	−45 (5)	N4—C5—C6—N3	−6(2)
N11—Ni1—N3—C4	−98.9 (15)	C4—N3—C6—C5	1(2)
N1—Ni1—N3—C4	−1.2 (15)	Ni1—N3—C6—C5	−177.4 (17)
N5—Ni1—N3—C6	−95 (2)	C9—N5—C7—N6	5(3)
N7—Ni1—N3—C6	−13 (2)	Ni1—N5—C7—N6	−175.1 (16)
N9—Ni1—N3—C6	134 (4)	C9—N5—C7—C10	175 (2)
N11—Ni1—N3—C6	79 (2)	Ni1—N5—C7—C10	−5(3)
N1—Ni1—N3—C6	177 (2)	C8—N6—C7—N5	−9(3)
N3—Ni1—N5—C7	90.6 (18)	C8—N6—C7—C10	−176 (3)
N7—Ni1—N5—C7	0.3 (18)	C7—N6—C8—C9	9(3)
N9—Ni1—N5—C7	−97.8 (18)	N6—C8—C9—N5	−7(3)
N11—Ni1—N5—C7	−63 (6)	C7—N5—C9—C8	1(3)
N1—Ni1—N5—C7	171.5 (19)	Ni1—N5—C9—C8	−179 (2)
N3—Ni1—N5—C9	−89 (3)	C12—N7—C10—N8	−2(2)
N7—Ni1—N5—C9	−179 (3)	Ni1—N7—C10—N8	171.0 (15)
N9—Ni1—N5—C9	83 (3)	C12—N7—C10—C7	178 (2)
N11—Ni1—N5—C9	118 (4)	Ni1—N7—C10—C7	−9(3)
N1—Ni1—N5—C9	−8(3)	C11—N8—C10—N7	2(3)
N3—Ni1—N7—C10	−93.7 (15)	C11—N8—C10—C7	−177 (2)
N5—Ni1—N7—C10	4.8 (15)	N5—C7—C10—N7	10 (3)
N9—Ni1—N7—C10	92.5 (16)	N6—C7—C10—N7	177 (2)
N11—Ni1—N7—C10	172.9 (16)	N5—C7—C10—N8	−170 (2)
N1—Ni1—N7—C10	−41 (4)	N6—C7—C10—N8	−3(5)
N3—Ni1—N7—C12	74 (3)	C10—N8—C11—C12	−2(3)
N5—Ni1—N7—C12	173 (3)	N8—C11—C12—N7	1(3)
N9—Ni1—N7—C12	−100 (2)	C10—N7—C12—C11	0(2)
N11—Ni1—N7—C12	−19 (3)	Ni1—N7—C12—C11	−168 (2)
N1—Ni1—N7—C12	127 (4)	C15—N9—C13—N10	−8(2)
N3—Ni1—N9—C13	−58 (4)	Ni1—N9—C13—N10	175.0 (13)
N5—Ni1—N9—C13	169.8 (15)	C15—N9—C13—C16	179.3 (18)
N7—Ni1—N9—C13	88.5 (16)	Ni1—N9—C13—C16	3(2)
N11—Ni1—N9—C13	−2.4 (14)	C14—N10—C13—N9	8(2)
N1—Ni1—N9—C13	−100.5 (14)	C14—N10—C13—C16	178 (2)
N3—Ni1—N9—C15	127 (4)	C13—N10—C14—C15	−4(2)
N5—Ni1—N9—C15	−5(2)	N10—C14—C15—N9	−1(2)
N7—Ni1—N9—C15	−87 (2)	C13—N9—C15—C14	6(2)
N11—Ni1—N9—C15	−177 (2)	Ni1—N9—C15—C14	−179.2 (16)
N1—Ni1—N9—C15	84 (2)	C18—N11—C16—N12	−1(2)
N3—Ni1—N11—C16	172.5 (15)	Ni1—N11—C16—N12	174.7 (13)
N5—Ni1—N11—C16	−34 (6)	C18—N11—C16—C13	−176.3 (19)
N7—Ni1—N11—C16	−95.9 (15)	Ni1—N11—C16—C13	−1(2)
N9—Ni1—N11—C16	1.7 (14)	C17—N12—C16—N11	1(2)
N1—Ni1—N11—C16	91.0 (15)	C17—N12—C16—C13	176 (2)
N3—Ni1—N11—C18	−15 (2)	N9—C13—C16—N11	−1(3)
N5—Ni1—N11—C18	139 (4)	N10—C13—C16—N11	−171 (2)
N7—Ni1—N11—C18	77 (2)	N9—C13—C16—N12	−176 (2)
N9—Ni1—N11—C18	175 (2)	N10—C13—C16—N12	15 (4)
N1—Ni1—N11—C18	−96 (2)	C16—N12—C17—C18	−1(2)
C3—N1—C1—N2	−1(2)	N12—C17—C18—N11	0(3)
Ni1—N1—C1—N2	168.2 (14)	C16—N11—C18—C17	0(2)
C3—N1—C1—C4	−177.3 (19)	Ni1—N11—C18—C17	−172.9 (18)
Ni1—N1—C1—C4	−8(2)	C21—N15—C19—O7	150 (7)
C2—N2—C1—N1	−2(3)	C20—N15—C19—O7	−51 (11)
C2—N2—C1—C4	173 (3)	C24—N16—C22—O8	−153 (6)
C1—N2—C2—C3	5(3)	C23—N16—C22—O8	0(10)
C1—N1—C3—C2	5(3)	C27—N17—C25—O9	−156 (8)
Ni1—N1—C3—C2	−160 (2)	C26—N17—C25—O9	18 (11)

### Hydrogen-bond geometry (Å, °)

**Table:**

*D*—H···*A*	*D*—H	H···*A*	*D*···*A*	*D*—H···*A*
N2—H2···O5^i^	0.86	2.19	2.93 (2)	144.
N2—H2···O4^i^	0.86	2.30	2.98 (2)	136.
N4—H4···O1^ii^	0.86	1.91	2.77 (2)	176.
N4—H4···O3^ii^	0.86	2.45	3.04 (3)	127.
N6—H6···O6	0.86	2.05	2.90 (3)	171.
N6—H6···O4	0.86	2.43	3.09 (2)	133.
N8—H8···O2^iii^	0.86	2.09	2.82 (3)	141.
N8—H8···O3^iii^	0.86	2.36	2.98 (2)	130.
N10—H10···O5^iv^	0.86	2.17	2.98 (3)	156.
N10—H10···O6^iv^	0.86	2.45	3.16 (2)	140.
N12—H12···O2^v^	0.86	2.29	2.97 (2)	136.

Symmetry codes: (i) *x*+1/2, *y*−1/2, *z*; (ii) *x*+1/2, −*y*+1/2, *z*+1/2; (iii) *x*, −*y*+1, *z*+1/2; (iv) *x*+1, *y*, *z*; (v) *x*+1, −*y*+1, *z*+1/2.
